# The Study on Sea Buckthorn (Genus *Hippophae* L.) Fruit Reveals Cell Division and Cell Expansion to Promote Morphogenesis

**DOI:** 10.3390/plants12051005

**Published:** 2023-02-22

**Authors:** Jing Zhao, Zhihua Zhang, Hongdan Zhou, Zengfu Bai, Kun Sun

**Affiliations:** College of Life Science, Northwest Normal University, Lanzhou 730070, China

**Keywords:** fruit development, cell division, cell expansion, *Hippophae rhamnoides* ssp. *sinensis*, *Hippophae neurocarpa*, *Hippophae goniocarpa*

## Abstract

Due to its unique flavor and high antioxidant content, the sea buckthorn (genus *Hippophae* L.) fruit is increasingly favored by consumers. Developing from the perianth tube, the sea buckthorn fruit varies greatly among species in both size and shape. However, the cellular regulation mechanism of sea buckthorn fruit morphogenesis remains unclear. This study presents the growth and development patterns, morphological changes, and cytological observations of the fruits of three *Hippophae* species (*H. rhamnoides* ssp. *sinensis*, *H. neurocarpa*, and *H. goniocarpa*). The fruits were monitored every 10–30 days after anthesis (DAA) for six periods in their natural population on the eastern margin of the Qinghai-Tibet Plateau in China. The results showed that the fruits of *H. rhamnoides* ssp. *sinensis* and *H. goniocarpa* grew in a sigmoid mode, while *H. neurocarpa* grew in an exponential mode under the complex regulation of cell division and cell expansion. In addition, cellular observations showed that the mesocarp cells of *H. rhamnoides* ssp. *sinensis* and *H. goniocarpa* were larger in the area with prolonged cell expansion activity, while *H. neurocarpa* had a higher cell division rate. Elongation and proliferation of the mesocarp cells were found to be essential factors affecting the formation of fruit morphology. Finally, we established a preliminary cellular scenario for fruit morphogenesis in the three species of sea buckthorn. Fruit development could be divided into a cell division phase and a cell expansion phase with an overlap between 10 and 30 DAA. In particular, the two phases in *H. neurocarpa* showed an additional overlap between 40 and 80 DAA. The description of the sea buckthorn fruit’s transformation and its temporal order may provide a theoretical basis to explore the growth mechanism of fruits and regulate their size through certain cultivation techniques.

## 1. Introduction

Sea buckthorn (genus *Hippophae* L. of family Elaeagnaceae), an ancient plant with modern value [1], is a perennial shrub or dungarunga with spiny branches. It exists naturally in the frigid regions of temperate and subtropical zones [2,3,4]. Sea buckthorn is dioecious, diploid (2n = 24), pollinated by wind, and has facultative parthenogenesis (FP). The distribution of sea buckthorn, aided by climate, soil, altitude, and other ecological factors, formed an abundant germplasm resource [5,6]. Of them, *H. Rhamnoides* ssp. *sinensis* is the most important and widely distributed species in China [7]. However, the agronomic potential of many *Hippophae* species remains underused or undisclosed. *H. neurocarpa* is a late-differentiated and most evolutive taxon of the group, distributed only in the high altitudes of the Qinghai-Tibet Plateau, and *H. goniocarpa* is a new taxon of the genus *Hippophae,* found only in a few areas of the Qilian County, Qinghai Province and the Songpan County, Sichuan Province [7,8]. They are also endemic species in China. 

The flowers of sea buckthorn are tiny, without a corolla, and appear before the leaves; male flowers have 2 sepals and 4 stamens, while female flowers have 2 sepals and 1 stigma, an inferior ovary, and yellow or orange pulpy fruits derived from the perianth tube. Each fruit contains one seed that is ovoid, shiny, and brownish-black in color [5,6]. Due to their unique taste and high medicinal value, the fruits of sea buckthorn are processed into drinks, jams, and dietary supplements and consumed worldwide [8,9]. Over the past few decades, researchers have focused on the various bioactive compounds present in fruits, including organic acids, phenols, flavonoids, and vitamins [9,10,11,12]. They have potential health-promoting benefits in humans due to their antioxidant and anti-inflammatory properties [13,14,15]. However, despite the growing interest in sea buckthorn, little is known about its fruit development and growth patterns. 

Potential fruit size is genetically controlled and is a qualitative trait that determines consumer preferences [16,17]. Fruit size is influenced by many factors, including water and nutrient availability and other environmental factors, such as climate, soil, and light [18]. Furthermore, it is also affected by anatomical features, including cell size, shape, and arrangement, cell wall thickness, cell-to-cell contact and volume of air space, and epidermal thickness [19,20,21,22]. In addition, different types of fruits show different developmental patterns. For example, most fruits, such as peaches [23], tomatoes [24], apples [25], and loquats [26], continue to engage in cell proliferation during early fruit development, with long-term cell expansion at later stages. However, the avocado pericarp continues to divide until before ripening [27]. 

Available information on sea buckthorn fruit focuses on either the fruit anatomy or the physicochemical properties of the mature drupes. Studies on the course of events leading to fruit growth and development are scarce. Therefore, this study aims to describe the variations in growth patterns and anatomical cytoarchitecture of three sea buckthorn species, namely *H. rhamnoides* ssp. *sinensis*, *H. neurocarpa,* and *H. goniocarpa*, and identify the key cellular program shift points to illustrate the coordination of cell division and cell expansion in controlling fruit morphogenesis. This study offers important information to understand the development and productivity of sea buckthorn fruits.

## 2. Results

### 2.1. General Observation

**Figure 1:**
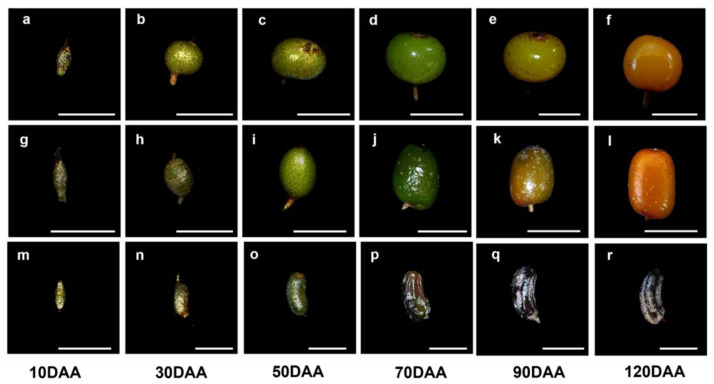
Morphological phenotypes of fruits of three species of *Hippophae* from full fruitlets at 10 DAA until the maturation (120 DAA). (**a**–**f**) *H. rhamnoides* ssp. *sinensis*; (**g**–**l**) *H. goniocarpa*; (**m**–**r**) *H. neurocarpa*; Bars = 5 mm.

**Figure 2:**
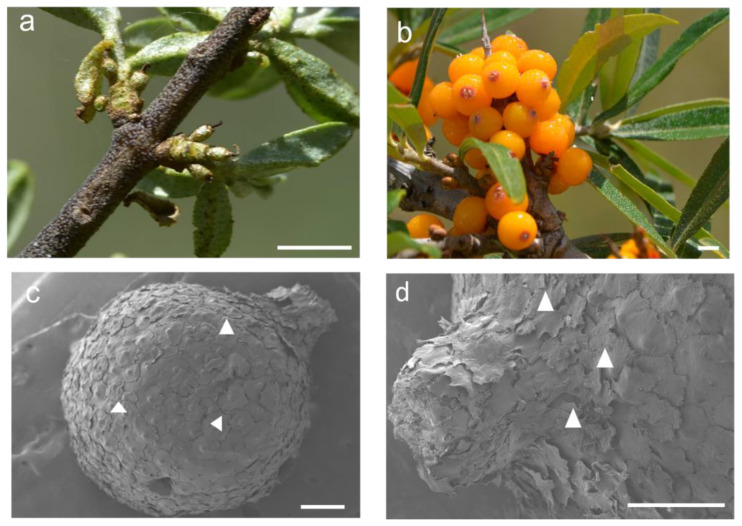
Morphological and microscopic images of *H. rhamnoides* ssp. *sinensis* fruit at 10 DAA and 120 DAA. (**a**) Fruits at 10 DAA, bar = 5 mm; (**b**) Fruits at 120 DAA, bar = 5 mm; (**c**) SEM micrographs show the fruit surface at 10 DAA, bar = 500 µm; (**d**) SEM micrographs show the fruit surface at 120 DAA, bar = 500 µm. The white triangle indicates the peltate trichomes of the perianth tube.

The dea buckthorn fruit was set 10 days after pollination, and the perianth tube became part of the developing fruit. There was no clear differentiation in the newly formed fruits at 10 days after anthesis (10 DAA) of the three *Hippophae* species (Figure 1a,g,m). The fruits were green in color and covered with glossy peltate trichomes that were gradually shed during the fruit’s development. Eventually, only a few of them persisted over the mature fruits (Figure 2). As the fruits matured (90–120 DAA), the peel color of the *H. rhamnoides* ssp. *sinensis* fruit changed from green to yellow. It was also single-seeded. From a tactile perspective, the fruits were near-spherical in shape with a soft peel that could be crushed easily. The fruits of *H. goniocarpa* were elliptical in shape and orange in color. The fruits of *H. neurocarpa* were long, cylindrical, and bent, having five grooves on the brown color rind (Figure 1f,l,r).

### 2.2. Fruit Growth Pattern

**Figure 3:**
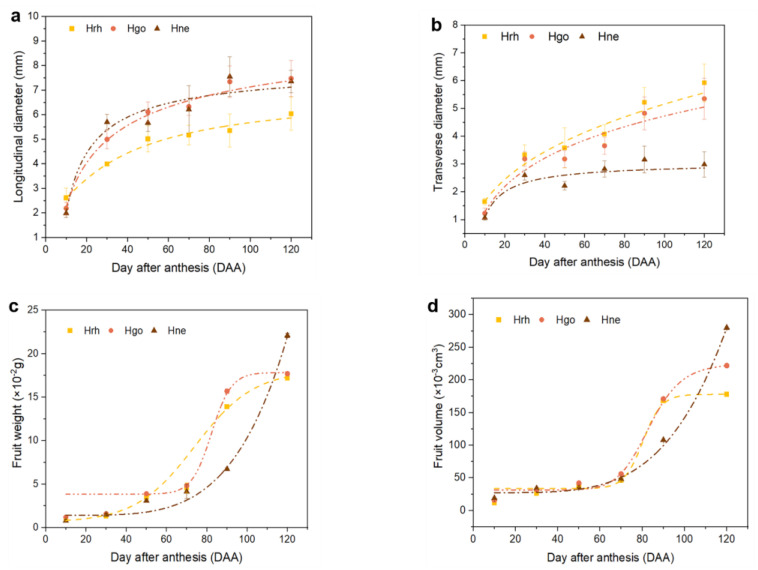
The fruit characteristics of three species of *Hippophae.* (**a**) Longitudinal diameter; (**b**) Transverse diameter; (**c**) Fruit weight (**d**) Fruit volume. Hrh *H. rhamnoides* ssp. *sinensis*; Hgo *H. goniocarpa*; Hne *H. neurocarpa.* Error bars represent the standard deviation (SD). (n = 50 fruits).

The fruit growth of H. rhamnoides ssp. *sinensis*, *H. goniocarpa*, and *H. neurocarpa* was defined by morphological changes, including transverse diameter, longitudinal diameter, fresh fruit weight, and volume (Figure 3). These morphological traits fitted well to the logistic model, and each quadratic coefficient was greater than a 0.9 regression coefficient (Table 1). Although these *Hippophae* fruits exhibited diverse fruit size and shape (Figure 1), the growths of their transverse and longitudinal diameter showed similar sigmoid growth curves, exponentially increasing at 0 to 50 DAA and continuously growing with a lower rate from 50 to 120 DAA (Figure 3a,b). Moreover, the fresh fruit weight and volume of the three *Hippophae* fruits also increased with an increase in the longitudinal and transverse diameters (Figure 3c,d). Fruit weight of *H. rhamnoides* ssp. *sinensis* and *H. goniocarpa* showed a sigmoidal growth trend, while *H. neurocarpa* fruit showed exponential growth. Additionally, in both *H. rhamnoides* ssp. *sinensis* and *H. goniocarpa*, the linear portion of the curves corresponded to a phase of intense development from 70 to 90 DAA, after which both fruit weight and volume remained steady while the maturation events occurred in fully expanded fruits. 

**Figure 4:**
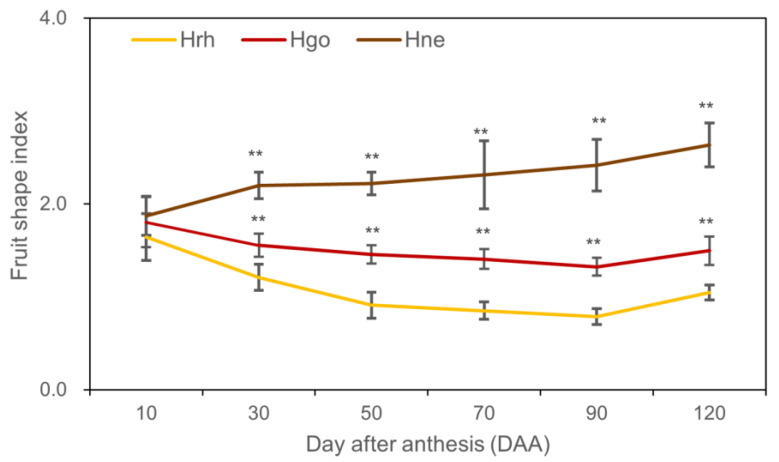
Fruit shape index of three species of *Hippophae.* Hrh *H. rhamnoides* ssp. *sinensis*; Hgo *H. goniocarpa*; Hne *H. neurocarpa.* Error bars represent the standard deviation (SD). “**” indicates significant differences (*p* < 0.01) among three species of *Hippophae* at the same period. (n = 50 fruits).

During early development (10 DAA), there was no significant difference in the fruit shape index among the three species (Figure 4). When fruits reached 30 DAA, the fruit shape index of *H. rhamnoides* ssp. *sinensis* and *H. goniocarpa* decreased gradually until the fruits reached 50 DAA. *H. rhamnoides* ssp. *sinensis* approached 1, while *H. goniocarpa* was close to 1.5. However, the fruit shape index of *H. neurocarpa* increased along with fruit development and approached 2.5 when the fruit reached 120 DAA (Figure 4).

### 2.3. Cellular Structure Changes

**Figure 5:**
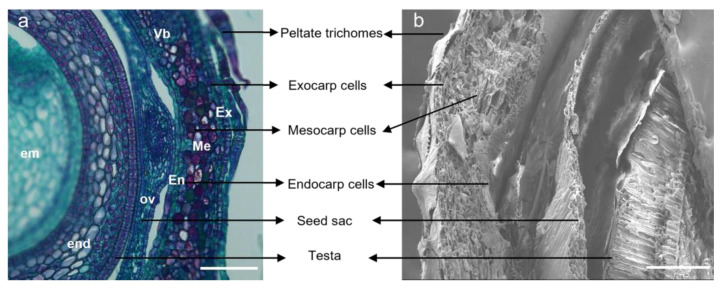
LM and SEM micrographs of *H. rhamnoides* ssp. *sinensis* fruit at 10 DAA. (**a**) LM micrograph shows transversal section; bar = 100 μm; (**b**) SEM micrograph shows transversal section bar = 200 μm. The perianth tube eventually develops into the fleshy part of the fruit, and the ovary gradually shrinks and forms a thin papery covering, known as seed sac, over the mature seed. Ex Exocarp cells; Me Mesocarp cells; En Endocarp cells; Vb Vascular bundle; Ov Ovary; End endosperm; Em Embryo.

Cellular changes in the fruits of the three *Hippophae* species were represented by tissues taken from 10 to 120 DAA. The pericarp of sea buckthorn that developed from the perianth tube was specialized into three parenchyma cell layers, namely the exocarp, mesocarp, and endocarp (Figure 5). The 1-2-layered exocarp was covered by peltate trichomes, with a few stomatal apparatuses. The mesocarp was the fleshy part of the hypanthium, with 5 to 8 layers of cell thickness. The 1-2-layered endocarp was located in the innermost of the pericarp cells. In addition, the size of the parenchyma cells in the mesocarp was larger compared with those in the exocarp and the endocarp, as observed in the transverse section of the fruit (Figure 5).

**Figure 6:**
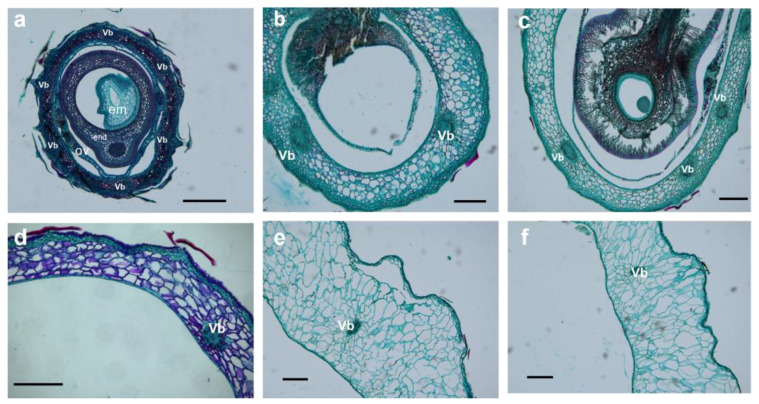
LM micrographs show transversal sections of *H. rhamnoides* ssp. *sinensis* fruit throughout six stages (10 DAA–120 DAA) of growth and development. (**a**) Cellular structure at 10 DAA. Six vascular bundles were arranged circularly on the side of mesocarp cells; (**b**) Cellular structure at 30 DAA; (**c**) Cellular structure at 50 DAA; (**d**) Cellular structure at 70 DAA; (**e**) Cellular structure at 90 DAA; (**f**) Cellular structure at 120 DAA. Bars = 250 μm. Vb Vascular bundle; Ov Ovary; End endosperm; Em Embryo.

**Figure 7:**
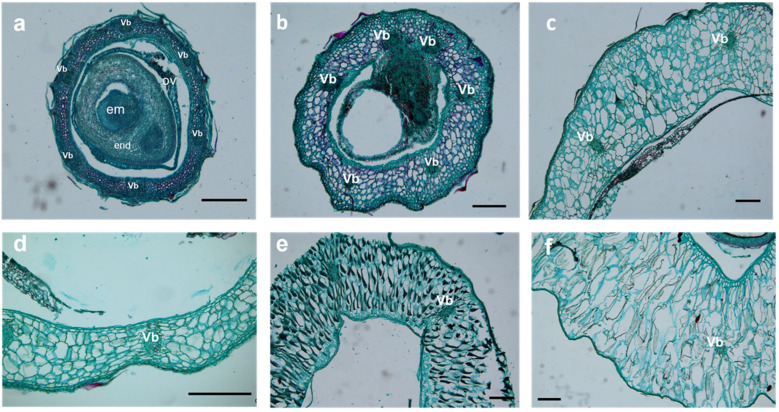
LM micrographs show transversal sections of *H. goniocarpa* fruit throughout six stages (10 DAA–120 DAA) of growth and development. (**a**) Cellular structure at 10 DAA. Six vascular bundles were arranged circularly on the side of mesocarp cells; (**b**) Cellular structure at 30 DAA; (**c**) Cellular structure at 50 DAA; (**d**) Cellular structure at 70 DAA; (**e**) Cellular structure at 90 DAA; (**f**) Cellular structure at 120 DAA. Bars = 250 μm. Vb Vascular bundle; Ov Ovary; End endosperm; Em Embryo.

**Figure 8:**
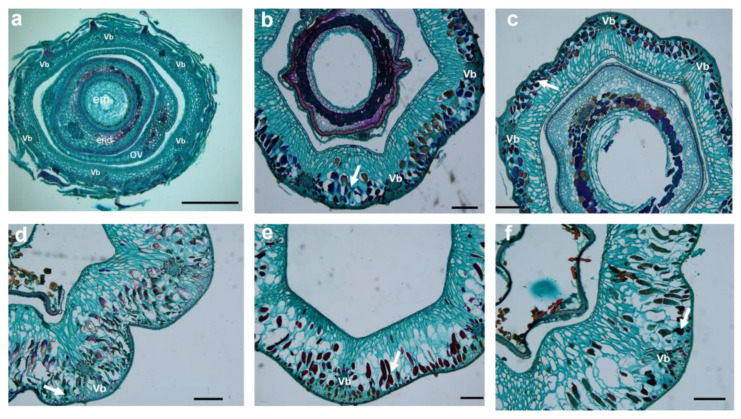
LM micrographs show transversal sections of *H. neurocarpa* fruit throughout six stages (10 DAA–120 DAA) of growth and development. (**a**) Cellular structure at 10 DAA. Six vascular bundles were arranged circularly on the side of mesocarp cells; (**b**) Cellular structure at 30 DAA. Some cells accumulated phenolics, as indicated by the arrow; (**c**) Cellular structure at 50 DAA; (**d**) Cellular structure at 70 DAA; (**e**) Cellular structure at 90 DAA; (**f**) Cellular structure at 120 DAA. Bars = 250 μm. Vb Vascular bundle; Ov Ovary; End endosperm; Em Embryo.

**Figure 9:**
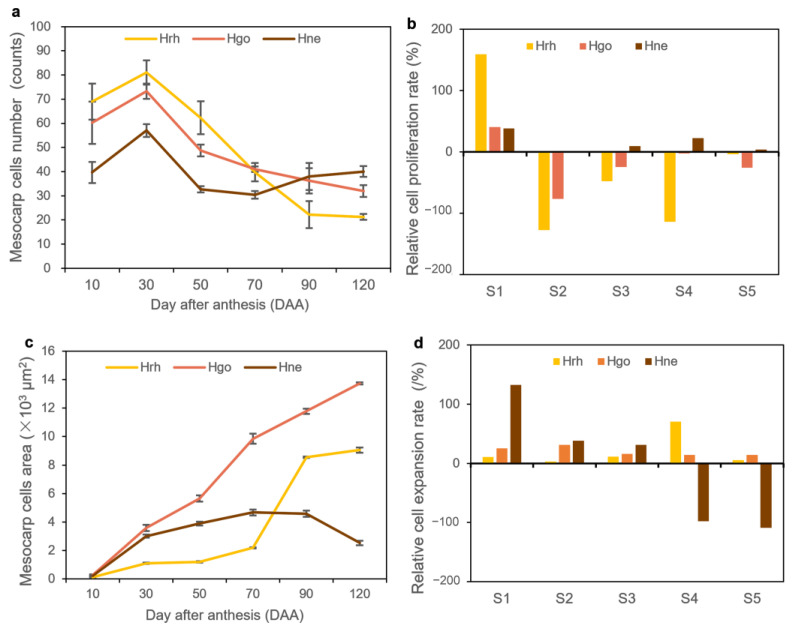
Cell proliferation and cell expansion during fruit morphogenesis of three species of *Hippophae*. (**a**) Changes of number in mesocarp cells; (**b**) The relative cell proliferation rate; (**c**) The cell area of the mesocarp cells (**d**) The relative cell expansion rate. Hrh *H. rhamnoides* ssp. *sinensis*; Hgo *H. goniocarpa*; Hne *H. neurocarpa.* Error bars represent the standard deviation (SD).

Parenchyma cells in the mesocarp of S1 (stage of 10 to 30 DAA) were small, irregular, and tightly packed. Some of the parenchyma cells were specialized to form six vascular bundles, arranged on the medial side of the mesocarp in a circular pattern (Figure 6a,b, Figure 7a,b, and Figure 8a,b). After fertilization, the number of mesocarp cells in *H. rhamnoides* ssp. *sinensis* and *H. goniocarpa* continued to increase until 30 DAA (Figure 9a). The relative cell proliferation rate also showed that most cell numbers of the three species were produced during early development (Figure 9b).

After 30 DAA, the mesocarp became 8-10-cell layers thick, accumulating phenolics, oils, polysaccharides, and proteins. However, their specific contents still needed to be measured further. As the fruits matured (from 90 to 120 DAA), parenchyma cells were found to be more irregular and vacuolated, along with enlarged intercellular spaces. The visibility of vascular bundle tissues was reduced (Figure 6e,f, Figure 7e,f and Figure 8e,f). The cells of the three *Hippophae* species fruits began to enlarge approximately 10 DAA until 70 DAA, after which the cell size of *H. rhamnoides* ssp. *sinensis* and *H. goniocarpa* entered a fast cell expansion period, with a quick cell area increase from 70 to 120 DAA and growing to a final size at 120 DAA (Figure 9c). During the same period, the mesocarp cell area of *H. neurocarpa* decreased due to water loss in the fruit, whereas the number of cells increased until 120 DAA (Figure 9a,c). An analysis of the relative cell expansion and proliferation rates of the three *Hippophae* species fruits in Figure 9b,d revealed that, compared with cell division, *H. rhamnoides* ssp. *sinensis*, *H. neurocarpa,* and *H. goniocarpa* fruits consumed more time for cell expansion during the whole fruit development. The longer time required for cell enlargement led to the finding that the mesocarp cells of mature fruits were almost ten to one hundred times larger compared with the cells of the perianth tube during anthesis.

## 3. Discussion

Fruit size is an attractive phenotypic trait associated with commercial value. The remarkable diversity of fruit size makes sea buckthorn a good biological system to study the genetic basis and regulating mechanisms in fruit development. Cell division and cell expansion usually directly influence the formation and development of final fruit sizes. The contribution of these two mechanisms to fruit growth can differ between species or cultivars. In melon and pumpkin, differences in the duration and the degree of cell expansion were observed [28,29]. Similarly, differences in the duration of cell divisions post-bloom were observed in different varieties of blueberry [30]. In this study, we investigated the dynamic fruit size changes in *H. rhamnoides* ssp. *sinensis*, *H. goniocarpa,* and *H. neurocarpa* from the morphology and cellular level aspects, and aimed to identify the key cellular program shift points to illustrate the coordination of cell division and cell expansion in controlling fruit morphogenesis. 

### 3.1. Fruit Growth and Development in Hippophae L.

In *Hippophae* spp., the perianth tube contributes to the formation of the fleshy layer. Furthermore, the ovary wall develops into a thin papery pericarp called the seed capsule, either separated from or attached to the seed coat [6,7,31]. The fruit type in *Hippophae* is not a true berry, as its description does not fully fit into the botanical classification of any fruit [32,33]. In a detailed investigation of *H. rhamnoides* cv., Harrison and Beveridge [34] suggested that the fruit of sea buckthorn should be described as achene, because the presence of a single seed in the fruit and indehiscent attachment of seed from a single point and development from a unilocular ovary are consistent with an “achene”. However, the achene by definition does not have a well-differentiated seed coat [33], while in sea buckthorn, the seed coat possesses a distinguishable testa. Additionally, another typical feature of achene is the dry nature of the fruit, which contrasts with the fleshy fruits in sea buckthorn. The fruit of sea buckthorn is similar to *Elaeagnus angustifolia* L., and therefore better described as “acrosarcum” (perianth tube forming fleshy parts and seed embedded in fleshy pulp) or “pseudo drupe” (the pericarp lacks a stony endocarp) [6,35]. Mangla et al. [6] also believed that the fruit of sea buckthorn might be appropriately described as a pseudo-drupe.

The fruits of sea buckthorn are used in a variety of medicinal and nutritional products. Fruits are collected from the female plants in the wild. It is known that the species fruits profusely and also propagates by forming root suckers, in a case very similar to *Paspalum grasses* [36] and *Urochloa* [37]. The occurrence of diverse reproductive pathways assures the possibility of generation of novel genotypes through sexuality, while apomictic reproduction maintains adaptive genotypes and ensures reproduction in the absence of pollination [6].

At present, there is a question as to whether *Hippophae* fits better into a single or double S model. In this study, other than the pattern followed by *H. neurocarpa*, *H. rhamnoides* ssp. *sinensis* and *H. goniocarpa* followed a single S model similar to that of other fruits such as apples [18,25] and loquats [26,38]. *H. neurocarpa* fruit displayed a single sigmoid curve where length, diameter, fresh weight, and volume increased exponentially as the fruit developed from 10 to 120 DAA. Similar growth patterns were also found in *Eugenia stipitate* [39], Rastali banana [40], and *Carissa congesta* [41]. The result also revealed that the fruit shape index of *Hippophae* varied with time. At the beginning of fruit set, the fruit shape index was high, giving *H. rhamnoides* ssp. *sinensis* and *H. goniocarpa* fruit an elongated shape. As the fruit grew, the elongation gradually slowed down while transverse diameter increased rapidly. When the fruit reached 90 DAA, the fruit of *H. rhamnoides* ssp. *sinensis* appeared almost roundish in shape, and the shape of the *H. goniocarpa* fruit was ellipsoidal. The longitudinal diameter growth of *H. neurocarpa* was higher than the transverse diameter growth, so the fruits of *H. neurocarpa* were cylindrical at 90 DAA. During development, fruit becomes the sink organ to accumulate photosynthate products from photosynthesis, such as sugar and water [42]. Thus, this is the major contributor to the increase in length, diameter, weight, and volume in *Hippophae* fruit.

### 3.2. Effects of Cell Division and Cell Extension on the Fruit Size of Hippophae L.

Cell division and cell expansion during fruit development are the key parameters affecting the final fruit size [43,44]. Cell observations showed that cell division increased rapidly shortly after flowering and fertilization. Compared with the early stage of development, there was no significant cell number increase in the mature fruits of the three *Hippophae* species. Cell number also increased after anthesis in loquats [26,45] and apples [18,25], and the number of cortex cells in a mature apple increased to five or more times that of receptacle cells during anthesis [25]. A large amount of variation in the cell number of the cortex might be an important reason for the larger size of the apple, especially the fleshy part [26]. 

In general, a combination of a greater cell division capacity and an enhanced degree of cell enlargement are involved in the increase in the fruit size [27]. Cell division continues in the skin of an avocado until shortly before ripening [46], whereas other fruits, such as sweet cherries [47], tomatoes [24], and apples [18,25], engage in cell proliferation early in fruit development, with long expansion until mature. In banana, it was demonstrated that “the maximum fruit filling rate is the product of pulp cell number and maximum cell filling rate” [48]. The investigations above show that cell division and cell enlargement might function individually or may cooperate with one another to determine the fruit size. In our study, compared with cell division, more time was spent on cell expansion in the sea buckthorn fruit during growth, which made the size of the pulp cells in the middle to late fruit development stages about ten to one hundred times bigger when compared with the size of the cells in the early fruit-setting stage or flower development stage. However, in *H. neurocarpa,* cell division was still active in the middle and late stages of fruit development, which could be due to the large number of cells required to make up for the small cell size at the maturity stage.

### 3.3. A Model of Cell Regulation in Fruit Development of Hippophae L.

**Figure 10:**
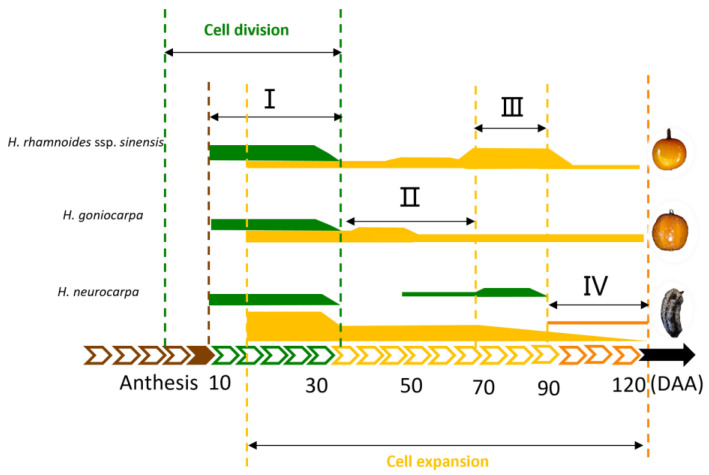
Cellular scenario for three species of *Hippophae* fruit development. Green lines represent cell division, yellow lines represent cell expansion. The different line widths represent the strength of cell division or expansion. I. Cell proliferation; II. Slow growth stage (or fruit hard core stage); III. Rapid growth stage; IV. Fruit ripening.

Based on the observations of the main morphological indexes of fruit growth and development, we established a preliminary model of cell regulation in fruit development in three species of *Hippophae*, as shown in Figure 10. The whole fruit growth process can be divided into a cell division phase and a cell expansion phase, with an overlap between 10 and 30 DAA. In particular, the two phases in *H. neurocarpa* showed an additional overlap between 40 and 80 DAA. Based on the degree of cell division and the intensity of cell expansion, fruit formation was divided into four stages, including cell proliferation, slow growth stage (or fruit hardcore stage), rapid growth stage, and fruit ripening. 

## 4. Materials and Methods

### 4.1. Plant Materials

The fruiting trees of three *Hippophae* species (*H. rhamnoides* ssp. *sinensis*, *H. neurocarpa* and *H. goniocarpa*) was monitored, from May to September 2021, in adult individuals of a natural hybrid zone of sea buckthorn in the eastern margin of the Qinghai-Tibet Plateau of Qilian County, Qinghai Province, China (38°15′ N, 100°16′ E). The average annual precipitation is 415.5 mm, and the average annual temperature is −1 °C.

Ten plants from the native population of each *Hippophae* species were selected based on their overall homogeneity with respect to canopy size and matching phenological stages of the plant and inflorescence. During the flowering season, inflorescence development was closely monitored. Samples of fruits were collected starting 10 days after anthesis (10 DAA) until 120 days after anthesis (120 DAA), when the fruits were commercially ripened. Part of the fruits were used for growth kinematics inspection, while the others were used for sampling.

### 4.2. Methods

#### 4.2.1. Fruit Characteristics of Sea Buckthorn during Development

Within 24 h of harvest, fruit longitudinal diameter was measured from the fruit stem end to the proximal end of each fruit by using a digital vernier caliper (LR44 AG13, Hengliang, China). Furthermore, the transverse diameter was measured at two opposite sides of mid region. The mean values of the fruit diameter were then calculated. The fresh weight was determined by using an electronic balance. The volume of fruits was estimated by immersing the fruit in a water-filled measuring cylinder (25 mL) and measuring the amount of water displaced by the complete immersion. The fruit shape index was calculated according to the following equation: fruit shape index = longitudinal diameter/transverse diameter. Moreover, at least fifty fruits were measured per repetition at each time point.

#### 4.2.2. The Microstructure of Fruits at Different Development Stages

For each sample point, three different fruits were picked and used for paraffin section analysis with the following procedure. First, the fruits were immediately fixed in FAA (70% ethanol:formaldehyde:acetic acid with a volume ratio of 90:5:5) for 24 h, dehydrated through a series concentration of ethanol (70, 85, 95, and 100%, each for 1 h, respectively), transferred to xylene for 2 h (replace with new xylene after 1 h), and embedded in paraffin. Furthermore, longitudinal and cross sections with 10 μm thickness were cut using a rotary microtome (Leica RT2235, Barcelona, Spain). The sections were stained with 0.1% safranin O and Fast Green solutions and mounted using Canada balsam. Lastly, the well-stained sections were sealed with resin and coverslips and photographed (Leica DM6 B, Leica Microsistemas S.L.U., Barcelona, Spain). 

For the SEM study, the samples were vaccumed and post-fixed in FAA for 24 h. Samples were then subjected to dehydration process in an increasing gradient of ethanol series, 30 min in each concentration. The samples were then dried in a SCIENTZ-10N vacuum freeze dryer (SCIENTZ, Ningbo, China), mounted on metal stubs, and sputter coated(Vision Precision Instruments, Beijing, China) in gold. Prepared samples were observed under high vacuum with thermal field emission scanning electron microscopy (Carl Zeiss AG, Oberkochen, Germany).

The sea buckthorn fruit is a pseudo-drupe, and for the convenience of description, the fruit pericarp cells were roughly divided into exocarp, mesocarp, and endocarp cells, from the exterior to the core cells, in this study. The anatomical parameters of *H. rhamnoides* ssp. *sinensis*, *H. neurocarpa,* and *H. goniocarpa* fruits at different stages of development were measured using Image J software (https://imagej.net/ij/index.html/, accessed on 7 February 2022) [49]: the cell area and cell number of mesocarp cells were measured. The relative cell proliferation rate and relative cell expansion rate were determined from the cell number and cell area data as follows. Relative growth (%) = (parameter value of a period/parameter value of fruit ripening period − parameter value of previous period/parameter value of fruit ripening period) × 100 [50]. The period from 10 to 30 DAA was defined as S1 (stage 1), and in the same manner, the periods from 30 to 50 DAA, 50 to 70 DAA, 70 to 90 DAA, and 90 to 120 DAA were set as S2, S3, S4, and S5, respectively.

### 4.3. Statistical Analysis

All parameters were subjected to the analysis of variance (ANOVA) using SPSS 20.0, with means being analyzed by regression analyses at *p* < 0.05 using the statistical software Origin 2020. Data in the graphs are mean ± SD.

## 5. Conclusions

The growth characteristics and cellular developmental properties of *H. rhamnoides* ssp. *sinensis*, *H. goniocarpa*, and *H. neurocarpa* were observed throughout their developmental stages. The results showed that the fruits of *H. rhamnoides* ssp. *sinensis* and *H. goniocarpa* grew in a single sigmoid mode, while *H. neurocarpa* grew in an exponential mode under the complex regulation of cell division and cell expansion. The results of cellular observations showed that the mesocarp cells of *H. rhamnoides* ssp. *sinensis* and *H. goniocarpa* were larger in cell area, with prolonged cell expansion activity, whereas *H. neurocarpa* had a higher cell division rate. Elongation and proliferation of the mesocarp cells were essential factors affecting fruit morphology. Finally, a preliminary cellular scenario for three species of sea buckthorn was established for fruit morphogenesis. Fruit development was divided into a cell division phase and a cell expansion phase, with an overlap between 10 and 30 DAA. These two phases in *H. neurocarpa* overlapped once again between 40 and 80 DAA. 

This study provides a theoretical basis to explore the growth mechanism of fruits and regulate their size through certain cultivation techniques. Further studies are required to understand the genetic basis of the growth pattern and to study the key genes regulating cell division and expansion, speed up the development of the *Hippophae* fruit, and improve the quality of the molecular breeding technology.

## Figures and Tables

**Table 1:** Logistic models fitted for longitudinal diameter, transverse diameter, fruit weight and volume against 10 until 120 DAA of three species of *Hippophae* fruit according to R^2^.

Species	Dependent Variable	Logistic Models	R^2^
*H. rhamnoides* ssp. sinensis	longitudinal diameter (mm)	$y=53935.24622-\frac{53934.37583}{1+\left(x/9991829.9436\right)0.8518}$	0.95192
	transverse diameter (mm)	$y=116.59846-\frac{116.8177}{1+\left(x/278566.0783\right)0.3595}$	0.97472
	fruit weight (×10^−2^ g)	$y=19.66319-\frac{18.68195}{1+\left(x/76.36193\right)4.33979}$	0.99241
	fruit volume (×10^−3^ cm^3^)	$y=285152.48918-\frac{285133.8871}{1+\left(x/2129.47093\right)2.60375}$	0.99669
*H. goniocarpa*	longitudinal diameter (mm)	$y=8.52238-\frac{1124.49485}{1+\left(x/0.00293\right)0.6324}$	0.90595
	transverse diameter (mm)	$y=366.52593-\frac{4.34431}{1+\left(x/1.42036\right)0.52308}$	0.94341
	fruit weight (×10^−2^ g)	$y=1.95051-\frac{6384.06956}{1+\left(1482.4658\right)0.51778}$	0.90155
	fruit volume (×10^−3^ cm^3^)	$y=225.7352-\frac{194.18527}{1+\left(x/80.956552\right)10.8082}$	0.99714
*H. neuvocarpa*	longitudinal diameter (mm)	$y=366.52593-\frac{4.34431}{1+\left(x/1.42036\right)0.52308}$	0.94016
	transverse diameter (mm)	$y=53935.24622-\frac{53934.37583}{1+\left(x/9991829.9436\right)0.8518}$	0.90831
	fruit weight (×10^−2^ g)	$y=1539194.821-\frac{1539193.4}{1+\left(x/1398.00226\right)4.57105}$	0.98673
	fruit volume (×10^−3^ cm^3^)	$y=4290.32753-\frac{4262.92621}{1+\left(x/221.58206\right)4.50811}$	0.99948

## Data Availability

The datasets generated during and/or analyzed during the current study are available from the corresponding author on reasonable request.
